# Trihydroxyethyl Rutin Provides Neuroprotection in Rats With Cervical Spinal Cord Hemi-Contusion

**DOI:** 10.3389/fnins.2021.759325

**Published:** 2021-11-18

**Authors:** Yapu Liu, Qi Liu, Zhou Yang, Rong Li, Zhiping Huang, Zucheng Huang, Junhao Liu, Xiuhua Wu, Junyu Lin, Xiaoliang Wu, Qingan Zhu

**Affiliations:** ^1^Division of Spinal Surgery, Department of Orthopaedics, Nanfang Hospital, Southern Medical University, Guangzhou, China; ^2^Department of Spinal Surgery, Second Affiliated Hospital of Luohe Medical College, Luohe, China

**Keywords:** trihydroxyethyl rutin, neuroprotection, spinal cord injury, microvascular, electrophysiology, behavior, histology

## Abstract

**Objective:** To investigate the neuroprotective effects of trihydroxyethyl rutin in rats with cervical spinal cord hemi-contusion.

**Methods:** Adult male Sprague–Dawley rats were subjected to hemi-contusion at a stroke depth of 1.2 mm, and then intraperitoneally injected with 50 or 100 mg/kg trihydroxyethyl rutin per day for 12 weeks (T50 and T100 groups, respectively). Changes in somatosensory evoked potentials (SEPs), motor evoked potentials (MEPs), and behavior were continuously monitored. At 12 weeks post-injury, immunohistochemical staining was performed to assess changes in cervical spinal cord microvascular morphology. Magnetic resonance imaging (MRI) scans were performed to examine end-stage injury in the cervical spinal cord, and Eriochrome cyanine-stained slices of spinal cord tissue were evaluated for injury.

**Results:** There were no significant differences in biomechanical parameters among the spinal cord injury, T50 and T100 rat groups. At 3 days-post-injury, there was a significant decrease in grip strength. At 12 weeks post-injury, grip strength recovery was significantly better in the T50 and T100 groups than in the injury group. Compared with the injury group, the total limb placement frequency was significantly higher in the T50 group at 2, 4, 6, 10, and 12 weeks post-injury and in the T100 group at 2, 6, 8, and 10 weeks post-injury. Ipsilateral SEPs and MEPs were dynamic, increasing in latency and decreasing in amplitude in the injury compared with sham group. MRI scanning demonstrated that the coronal, sagittal, and transversal lesion areas were smaller in the T50 and T100 groups than in the injury group. Microvascular density showed a greater reduction in the injury group compared with the T50 and T100 groups. Eriochrome cyanine staining showed that the ipsilateral side, residual parenchyma, and gray matter areas were larger in the T50 and T100 groups than in the injury group.

**Conclusion:** Trihydroxyethyl rutin exhibits robust neuroprotective effects, improving limb motor function and nerve electrophysiological parameters after spinal cord injury, maintaining microvascular density, and reducing the area of injury and degree of demyelination.

## Introduction

Spinal cord injury results from central nervous system trauma and seriously endangers human health. This major cause of disability imposes a heavy economic and social burden (Jain et al., 2015; Harman et al., 2018; Zhou et al., 2018). Various drugs and cell therapies have shown some potential in the treatment of spinal cord injury, and some clinical trials have shown promising results.

Trihydroxyethyl rutin is a flavonoid compound with the molecular formula C_3__3_H_4__2_O_19_. This compound contains three additional hydroxyethyl groups compared with the molecular structure of rutin (C_2__7_H_3__0_O_16_), which may confer various beneficial biological effects (e.g., protection against oxidation, protection against inflammation, reduction of capillary permeability, and reduction of intratissue bleeding). Zhai et al. (2016) reported a protective effect of rutin on nerves in the context of brain injury. Furthermore, rutin can promote neurological recovery after the onset of cerebral hemorrhage, cerebral infarction, Parkinson’s disease, and other central nervous system diseases (Hao et al., 2016; Li et al., 2017; Abdel-Aleem and Khaleel, 2018; Liu et al., 2018).

Considering the therapeutic effect of rutin on brain tissue injury, we presumed that it may also have a therapeutic effect in the context of spinal cord injury. However, there have been few studies regarding the neuroprotective effect of rutin in models of spinal cord injury; the potential underlying mechanism remains unknown (Wu et al., 2016; Song et al., 2018). The modified form of rutin, trihydroxyethyl rutin, has a robust chemical effect; the additional three hydroxyethyl groups greatly promote efficient drug absorption and utilization in the body, presumably enhancing their biological effects. To our knowledge, trihydroxyethyl rutin has been used clinically to protect brain tissue damage. However, the therapeutic effect and mechanism of trihydroxyethyl rutin in the context of spinal cord injury have not been reported. Here, we investigated whether trihydroxyethyl rutin exhibited neuroprotective effects in a model of spinal cord injury. The findings may help to establish new strategies for the treatment of spinal cord injury.

## Materials and Methods

### Cervical Spinal Cord Hemi-Contusion

In total, 48 adult male Sprague–Dawley rats (weighing 280–300 g) were purchased from the Southern Medical University Experimental Animal Centre (Guangzhou, China) and randomly divided into four groups: sham (*n* = 12), injury (*n* = 12), T50 (intraperitoneal administration of 50 mg/kg trihydroxyethyl rutin once daily, *n* = 12), and T100 (intraperitoneal administration of 100 mg/kg trihydroxyethyl rutin once daily, *n* = 12). Members of the sham group underwent laminectomy, as well as non-injurious contact of the injury probe to the exposed cord to control for the laminectomy; a “preparatory touch” was applied before actual injury in the other groups. Members of the spinal cord injury, T50, and T100 groups underwent laminectomy and spinal cord compression injury.

The cervical spinal cord hemi-contusion model was established using a previously published method (Liu et al., 2019a). An incision was made in the skin and muscle covering the C4–C6 cervical segment. A C5 laminectomy was performed without damaging the spinal cord during removal of the dorsal lamina; each rat was placed in a stereoscopic frame, with clamps on the spinous processes at the C4 and C6 segments to maintain stability during the injury procedure.

An electromagnetic probe (ElectroPuls E1000 testing machine; Instron, Canton, MA, United States) was oscillated in air and lowered to the surface of the exposed left spinal cord, targeted to the posterior median artery; upon activation, it caused a 1.2-mm displacement at a rate of 500 mm/s. Rats in the sham group were not subjected to the injury procedure. Subsequently, the rat was removed from the spinal frame and subjected to three layers of muscle closure, followed by skin closure. Each rat was administered intramuscular penicillin (400,000 units/day) to prevent infection.

### Behavior Testing

Two weeks before rats were subjected to spinal cord injury, they were trained to comfortably grip a mechanical detector and perform a forelimb asymmetry task. These tasks were specifically designed to measure basic and advanced motion. After the rats exhibited proficiency in these tasks, they were filmed for pre-injury data collection.

### Grip Strength Test

Grip strength was assessed as described previously (Aguilar and Steward, 2010). Briefly, the grip strength was tested using a YISIDA mechanical detector (YISIDA Technology Co., Qingdao, China). The longitudinal part of a 0.15-cm-diameter “T” type stainless steel strip was attached to the detector, and the side part was gripped by the rats. The experimenter’s left hand fixed the rat’s tail and torso; the right hand was placed in front of the rat’s torso to avoid interference from the right paw, thereby allowing the left front paw to move autonomously and flexibly. After the rat had grasped the test rod, the rod was moved backward in a smooth manner; the detector showed the maximum tensile force. Each rat was tested three times, and the maximum value was taken as the maximum grip strength.

### Forelimb Asymmetry Task

The forelimb asymmetry task, also known as the cylinder test, was used to measure changes in forelimb use during vertical exploration. Each rat was placed in a clear plastic cylinder and spontaneous exploratory behaviors were observed for 15 min. Slow-motion video playback was used to record instances in which the rat placed either its ipsilateral, contralateral, or bilateral limbs against the side of the cylinder during 20 weight-supported movements, in accordance with a previously published method (Schallert et al., 2000; Mondello et al., 2015). The proportion of ipsilateral limb placements relative to the total number of limb placements (averaged across all rats in each group) was calculated, and the values were compared across groups.

### Electrophysiological Evaluation

Forelimb somatosensory evoked potentials (SEPs) and motor evoked potentials (MEPs) were recorded preoperatively, and on postoperative days 3, 7, 14, 28, 42, 56, 70, and 84. Each rat was anesthetized with low-dose isoflurane; the head fur was then shaved and the skin was disinfected with 75% ethanol. A thermostatic heating pad was placed under the rat to maintain a body temperature of 37°C. Stimulation was delivered using a 16-channel neuromonitoring system (Yizhi Technology Co., Ltd., Hangzhou, China).

### Somatosensory Evoked Potentials

Continuous stimulation was used to generate a 5.3-Hz, 0.2-ms^2^ wave current for continuous stimulation of the median nerve of the forelimb; the current intensity was sufficient to induce slight forepaw contraction. The recording electrode was placed on the Cz–Fz axis of the cortex for recording. During a single test, 300 SEP signals were obtained to reduce the signal-to-noise ratio. The signal strength was amplified 100,000-fold by two amplifiers, and the waveform was filtered into 2–2,000-Hz waves using a filter device. SEP peak latency was measured from stimulus initiation to the peak of the first negative peak. Amplitude was calculated as the voltage difference between the first positive and first negative peaks (Forgione et al., 2014).

### Motor Evoked Potentials

Constant stimulation was used to generate MEPs. Electrodes were attached to the skull for anodal stimulation. A train of six constant current pulses (8–12 mA, 0.05 ms, 500 Hz) was delivered using a 16-channel neuromonitoring system. MEPs were recorded bilaterally from a single muscle group using two 1.2-cm needle electrodes placed approximately 1 cm apart. The recording electrodes were placed in muscle groups located on the mid-shaft of the forelimb (Morris et al., 2017).

### Magnetic Resonance Imaging

Twelve weeks after cervical spinal cord contusion, five rats in each group were randomly selected for 7.0 T MRI of the cervical spinal cord (ClinScan; Bruker, Berlin, Germany). To prevent respiratory movement and other factors from interfering with the scans, *in vitro* scanning of the cervical spinal cord tissue was performed. After each rat had been deeply anesthetized with Beuthanasia (phenytoin/pentobarbital mixture, 200 mg/kg), cold (4°C) heparin-saline (50 mg/500 mL) and 4% paraformaldehyde solution was perfused through the left ventricle. The cervical spinal cord tissue was removed and placed in 4% paraformaldehyde solution, and then immediately subjected to MRI scanning. The scanning parameters were as follows: repetition time/echo time, 2,200 ms/126 ms; image matrix, 256 × 256; and slice thickness, 1 mm (Li et al., 2015; Moon et al., 2018). Cervical spinal cord tissue was scanned in the coronal, sagittal, and transverse planes. The scans were stored in DICOM format and viewed using Sante DICOM Viewer software (Santesoft, Nicosia, Cyprus). Pseudo-color was added to cervical spinal cord images to increase the contrast; the damaged area was calculated using ImageJ software (National Institutes of Health, Bethesda, MD, United States).

### Three-Dimensional Reconstruction of Cervical Spinal Cord Microvessels

At the end of the trial (12 weeks after cervical spinal cord injury), one rat was randomly selected from each group for cervical spinal cord microvascular perfusion using a barium sulfate solution (medical gelatin, 5 g; barium sulfate, 20 g; physiological saline, 100 mL) (Liu et al., 2019a, b). Perfused cervical spinal cords were fixed to a small cystosepiment (U80810; outer diameter, 20 mm; inner diameter, 18.5 mm; height, 65 mm) for micro-computed tomography (CT) scanning (μCT 80; Scanco Medical AG, Switzerland) with an isotropic voxel length of 8 μm (55 kVp, 145 μA; integration time, 300 ms [averaged over two scans]). Each sample was embedded in the center of a sponge and then placed in a rotating focus X-ray tube for 360° scanning to obtain tomograms. Three-dimensional reconstruction images were obtained using the micro-CT analysis system.

### Immunohistochemistry

CD34 immunohistochemical staining for cervical spinal cord microvessels was performed 12 weeks after spinal cord injury, using a previously published method (Schallert et al., 2000). Rats were perfused with cold (4°C) heparin-saline (50 mg/500 mL) and 4% paraformaldehyde through the left ventricle. The spinal cords were collected, fixed in 4% paraformaldehyde for 24 h, and cryopreserved in phosphate-buffered saline containing 30% sucrose. The specimens were incubated at room temperature in optimal cutting temperature compound for 1 h, and then placed in the freezer (embedded in optimal cutting temperature compound) in the coronal position and rapidly frozen with dry ice. Specimen blocks were stored at −80°C before further processing. Spinal cords were cut coronally at intervals of 20 μm. The resulting sections were soaked with 3% hydrogen peroxide to block endogenous peroxidase activity and then incubated overnight with rabbit polyclonal anti-CD34 antibody (1:1,000; Bioworld Technology, St. Louis Park, MN, United States). Microvessel density was calculated using Weidner’s microvascular counting method (Weidner, 1996). The number of positive cells in each group was quantified in three random fields of normal tissue around the epicenter on the injury side.

### Eriochrome Cyanine Staining

Spinal cord tissue was dehydrated using a sucrose gradient (10, 20, and 30%). One-centimeter-long cervical spinal cord tissue was harvested with C5 segments as described above, embedded in optimal cutting temperature compound. Each block was cut using a cryostat into 20-μm-thick coronal sections and stored in a −80°C freezer. Tissue staining was performed on 10 slices at 200-μm intervals. Sections were then subjected to an ethanol dehydration gradient (water for 5 min, 55% ethanol for 5 min, 95% ethanol for 5 min, 100% ethanol for 5 min and incubation in xylene for 10 min). The steps were reversed to gradually rehydrate the tissue. Sections were then incubated in Eriochrome cyanine (Sigma, St. Louis, MO, United States) for 10 min, washed in water, and differentiated in 1% ammonium hydroxide (Sigma) for 30 s. The slices were dehydrated using the gradual ethanol gradient described above. The resulting slides were sealed with neutral resin gel and observed via microscopy. The lesion area was calculated using ImageJ; the largest part of the lesion was defined as the damage center. The total area of the damaged side, residual area, and residual gray matter area were outlined; the lesion area was then divided by the healthy tissue area to calculate the relative lesion area.

### Statistical Analysis

Statistical analysis was performed using SPSS Statistics (version 20.0; SPSS Inc., Chicago, IL, United States). All measurement results are expressed as mean ± standard error of the mean. Student’s *t*-test was used for comparison of independent variables between two groups. Two-way analysis of variance, with time as a repeated measure, was used to evaluate significant differences among all groups. *P* < 0.05 was considered statistically significant for all tests.

## Results

### The Biomechanical Parameters of the Spinal Cord Injury Model Were Consistent Among the Groups

**FIGURE 1:**
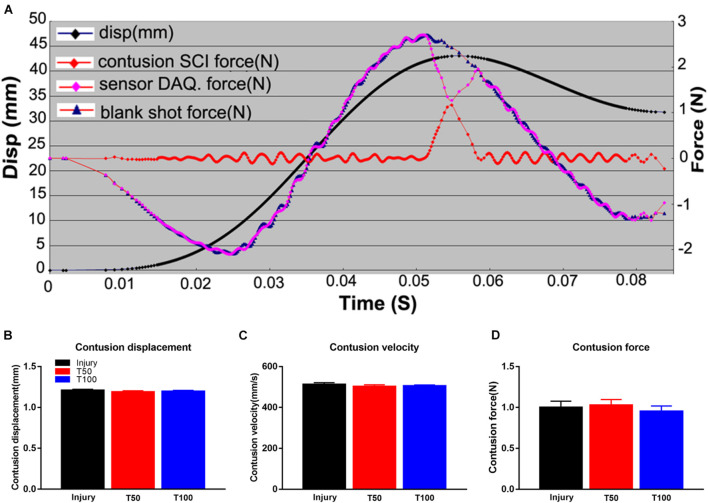
Biomechanical results of hemi-contusion. **(A)** Curve of displacement and velocity. **(B)** Contusion displacement comparison of each group. **(C)** Contusion velocity comparison of each group. **(D)** Comparison of the contusion forces of each group. There is no significant difference in biomechanical parameters between the groups.

The displacement depth and speed of the material testing machine were recorded by the engineer before creating the contusions, to ensure that the error was within 1%. In the injury group, the biomechanical results were as follows: displacement, 1.210 ± 0.003 mm; velocity, 507.700 ± 2.693 mm/s; and maximum load force, 1.000 ± 0.077 N. In the T50 group, the respective values were 1.189 ± 0.004 mm, 502.400 ± 2.408 mm/s and 1.027 ± 0.069 N; and in the T100 group, they were 1.197 ± 0.003 mm, 504.900 ± 1.366 mm/s and 0.953 ± 0.066 N. There were no significant differences in biomechanical parameters among the groups (Figure 1).

### Rats in the T50 and T100 Groups Presented Higher Superior Recovery in the Grip Strength Test

**FIGURE 2:**
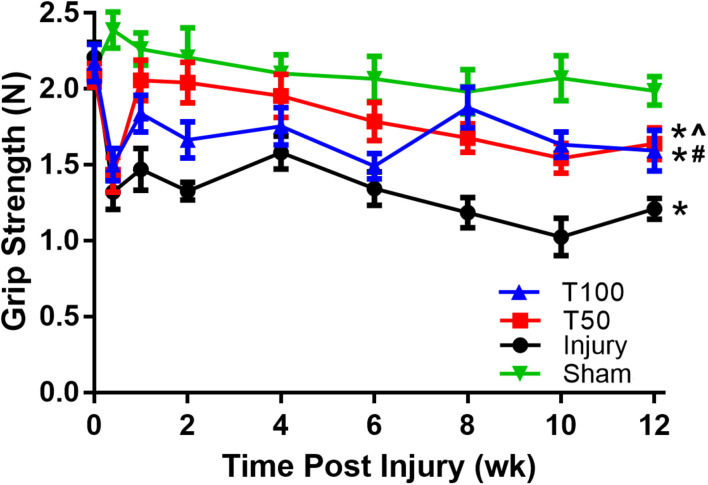
Grip strength test. The grip strength of the injury, T50, and T100 groups was significantly lower than that of the sham group. The overall recovery of the grip strength of the T50 group and the T100 group was significantly better than that of the injury group. *P < 0.05, comparison between each group and the sham operation group; ^#^*P* < 0.05, comparison between T50 group and injury group; ∧*P* < 0.05, comparison between T100 group and injury group.

The maximum grip strength of the forepaw was measured by a mechanical tester. Two weeks before contusion, the rats were trained to grasp the strip. In a quiet environment, the rats were prompted to resist the pulling movement and the maximum tensile force was recorded. On the third day after contusion, a significant decrease in grip strength was observed in each group. At 12 weeks after contusion, grip strength was significantly lower in the injury (1.21 ± 0.07 N), T50 (1.64 ± 0.11 N), and T100 (1.59 ± 0.14 N) groups compared with the sham group. Grip strength recovery was significantly superior in the T50 and T100 groups than in the injury group (*P* = 0.01 and *P* = 0.0391, respectively) (Figure 2).

### Rats in the T50 and T100 Groups Presented Higher Usage of Ipsilateral Forelimb in the Forelimb Asymmetry Task

**FIGURE 3:**
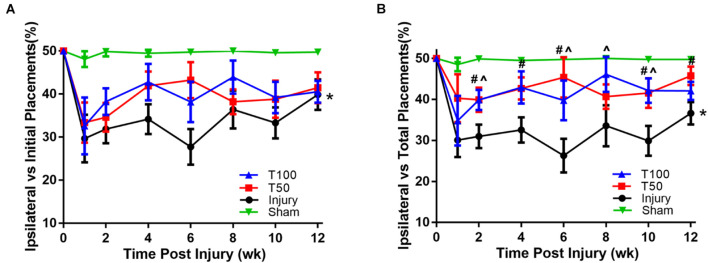
Forelimb asymmetry use task. **(A)** Frequency of initial placement. **(B)** Frequency of total placement. *P < 0.05, comparison between each group and the sham group; ^#^*P* < 0.05, comparison between T50 group and injury group; ∧*P* < 0.05, comparison between T100 group and injury group.

Normal rats generally use the bilateral forelimbs for climbing and touching. However, after cervical spinal cord injury, the injured side of the forelimb is used significantly less; the severity of cervical spinal cord injury and subsequent recovery were assessed herein based on this phenomenon. The frequency of initial limb placement in each group significantly decreased at 1 week after injury. At 12 weeks after contusion, the frequency of initial limb placement in the injury, T50, and T100 groups was 39.87 ± 3.56%, 41.5 ± 3.59%, and 40.56 ± 2.54%, respectively. The frequency of initial placement was significantly lower in the injury than sham group (*P* = 0.0479), and there was no significant pairwise difference between the T50 and injury group or T100 and injury group (*P* = 0.4901 and *P* = 0.8519, respectively). The frequency of total limb placement frequency in each group significantly decreased at 1 week after injury. At 12 weeks after contusion, the frequency of initial total placement in the injury, T50, and T100 groups was 36.63 ± 2.75%, 45.74 ± 2.26%, and 42.09 ± 2.23%, respectively. The frequency of total placement was significantly lower in the injury than sham group (*P* = 0.0113), and there was no significant pairwise difference between the T50 and injury group or T100 and injury group (*P* = 0.3254 and *P* = 0.8519, respectively) (Figure 3A). However, the total limb placement frequency was significantly higher in the T50 than injury group at 2, 4, 6, 10, and 12 weeks after contusion (*P* = 0.0428, *P* = 0.0217, *P* = 0.0069, *P* = 0.0336, and *P* = 0.0178, respectively), and the total limb placement frequency was significantly higher in the T100 than injury group at 2, 6, 8, and 10 weeks after contusion (*P* = 0.0280, *P* = 0.0452, *P* = 0.0498, and *P* = 0.0167, respectively) (Figure 3B).

### Rats in the T50 and T100 Groups Presented Shorter Latency and Higher Amplitude in Somatosensory Evoked Potentials Evaluation

**FIGURE 4:**
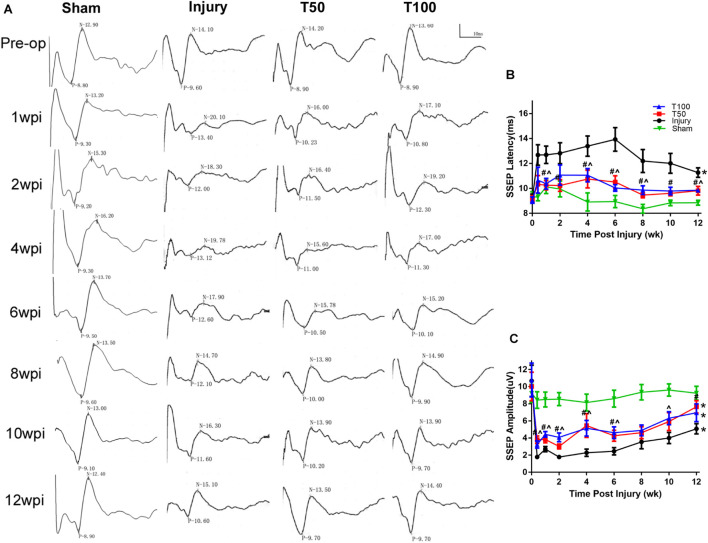
Somatosensory evoked potential (SEPs) results. **(A)** SEPs waveform from preoperative to postoperative week 1, 2, 4, 6, 8, 10, and 12. **(B)** SEPs latency; **(C)** SEPs amplitude. *P < 0.05, comparison between each group and the sham group; ^#^*P* < 0.05, comparison between T50 group and injury group; ∧*P* < 0.05, comparison between T100 group and injury group.

The signal of a single SEP was superimposed on multiple signals room the flexor of the forelimb to reduce the signal-to-noise ratio. The signal intensity was amplified 100,000-fold by two amplifiers, and the waveform was filtered into 2–2,000-Hz waves using a filter device. At the preoperative baseline, the latency and amplitude of the left forelimb SEPs in each group were generally similar (approximately 9.1 ms and 9.2 μV, respectively) among the groups. The latency was prolonged in each group at 3 days after injury. The latency at 12 weeks after injury was 11.27 ± 0.38, 9.98 ± 0.39, and 9.92 ± 0.17 ms, respectively, in the injury, T50, and T100 groups. The SEP latency was significantly longer in the injury than sham group (*P* = 0.0375), and there was no significant pairwise difference between the T50 and injury group or T100 and injury group. However, the latency was shorter in the T50 than injury group (*P* < 0.05) at all-time points from week 1–12; the latency was also shorter in the T100 than injury group (*P* < 0.05) at 1, 4, 8, and 12 weeks. The SEP amplitude decreased on the third day after injury in all groups. The SEP amplitude in the injury, T50, and T100 groups at 12 weeks after injury was 5.07 ± 0.60, 7.64 ± 0.71, and 6.95 ± 1.04 μV, respectively. The SEP amplitude was significantly lower in the injury, T50, and T100 groups than in the sham group (*P* < 0.0001, *P* = 0.0311, and *P* = 0.0178, respectively), and there was no significant pairwise difference between the T50 and injury group or T100 and injury group. However, the SEP amplitude was higher in the T50 than injury group (*P* < 0.05) at all-time points from 3 days to 6 weeks, and at 12 weeks; the SEP amplitude was higher in the T100 than injury group (*P* < 0.05) at all-time points from 3 days to 8 weeks, and at 10 weeks (Figure 4).

### Rats in the T50 and T100 Groups Presented Shorter Latency and Higher Amplitude in Motor Evoked Potentials Evaluation

**FIGURE 5:**
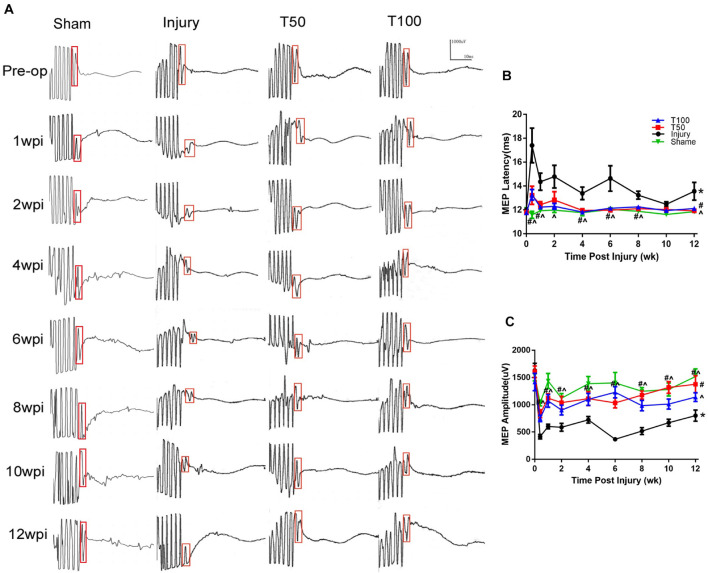
Motor evoked potentials (MEPs) results. **(A)** MEPs waveforms preoperative and postoperative. **(B)** MEPs latency; **(C)** MEPs amplitude. *P < 0.05, comparison between each group and the sham group; ^#^*P* < 0.05, comparison between T50 group and injury group; ∧*P* < 0.05, comparison between T100 group and injury group.

MEPs arise from a single stimulation of the cerebral motor cortex, and are received by subcutaneous cortical electrodes placed on the left and right forelimbs. At the preoperative baseline, the latency and amplitude of left forelimb MEPs in each group were generally similar (approximately 12 ms and 15,35 μV, respectively) among the groups. The latency was prolonged in each group at 3 days after injury. The latency at 12 weeks after injury was 13.56 ± 0.74, 11.92 ± 0.07, and 12.11 ± 0.16 ms in the injury, T50, and T100 groups, respectively. The MEP latency was significantly longer in the injury than sham group (*P* = 0.0023), and significantly shorter in the T50 and T100 groups than in the injury group (*P* = 0.0471 and *P* = 0.0250, respectively). The MEP amplitude decreased on the third day after injury in all groups. The MEP amplitude in the injury, T50, and T100 groups at 12 weeks after injury was 801.20 ± 101.70, 1,376.00 ± 157.00, and 1,141.00 ± 80.77 μV, respectively. The MEP amplitude was significantly lower in the injury than sham group (*P* < 0.0001), and was significantly higher in the T50 and T100 groups than in the injury group (*P* = 0.0079 and *P* < 0.0001, respectively) (Figure 5).

### Rats in the T50 and T100 Groups Presented Smaller Lesion Areas in Magnetic Resonance Imaging Scanning

**FIGURE 6:**
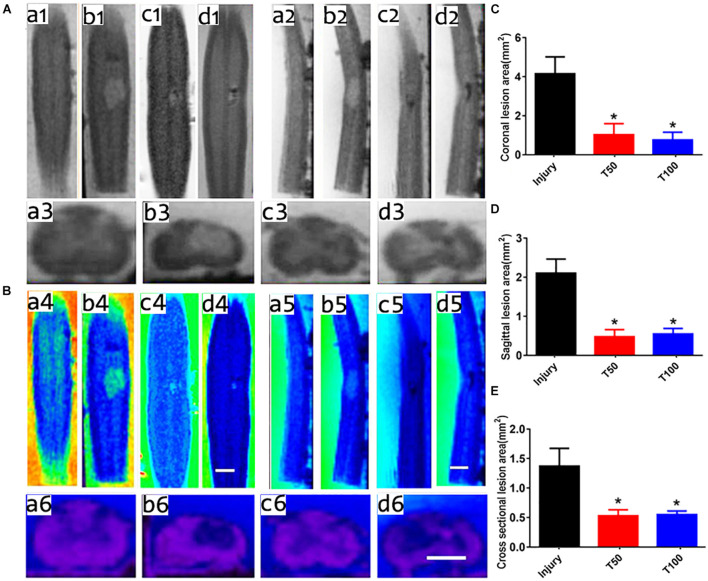
MRI scanning results. **(A)** (a1–d1) Coronal plane scanning for Sham group, injury group, T50 group and T100 group; **(A)** (a2–d2) sagittal plane scanning for Sham group, injury group, T50 group and T100 group; **(A)** (a3–d3) cross-sectional plane scanning for Sham group, injury group, T50 group and T100 group; **(B)** (a4–d4) color NIH coronal images for Sham group, injury group, T50 group and T100 group; **(B)** (a5–d5) color NIH sagittal images for Sham group, injury group, T50 group and T100 group; **(B)** (a6–d6) color NIH cross-sectional images for the Sham group, injury group, T50 group and T100 group; **(C)** lesions area of coronal plane; **(D)** lesions area of sagittal plane; **(E)** lesions area of cross-sectional plane. *P < 0.05, comparison between T50 group or T100 group and injury group. Scale bar = 2 mm.

At 12 weeks after injury, five rats were randomly selected from each group for cervical spinal cord MRI scanning. This examination revealed the boundary of the gray matter, which was useful for determining recovery from spinal cord injury in the coronal, sagittal, and transverse planes. The coronal lesion area in the injury, T50, and T100 groups was 4.19 ± 0.29, 1.03 ± 0.15, and 0.76 ± 0.12 mm^2^, respectively (Figures 6A1,B4,C); the sagittal lesion area was 2.11 ± 0.16, 0.48 ± 0.08, and 0.55 ± 0.06 mm^2^, respectively (Figures 6A2,B5,D); and the transversal lesion area was 1.37 ± 0.30, 0.53 ± 0.10, and 0.55 ± 0.06 mm^2^, respectively (Figures 6A3,B6,E). The coronal, sagittal, and transversal lesion areas were significantly smaller in the T50 and T100 groups than in the injury group (*P* < 0.05) (Figure 6).

### Rats in the T50 and T100 Groups Preserved Higher Vascular Density in Three-Dimensional Reconstruction

**FIGURE 7:**
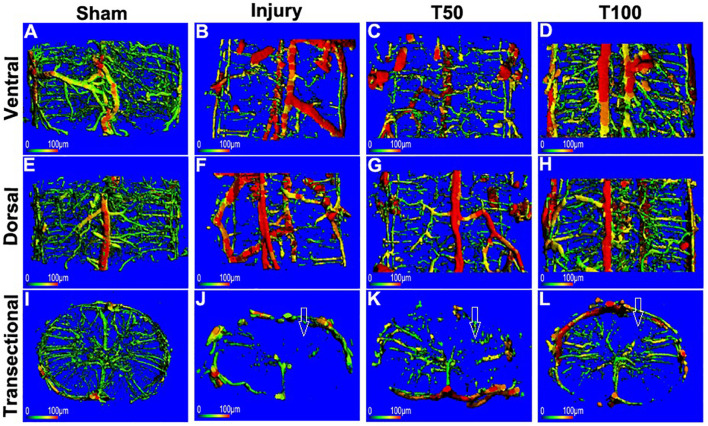
3D reconstruction of microvessels in the cervical spinal cord. **(A)** Ventral, **(E)** dorsal, **(I)** cross-sectional microvessels in Sham group; **(B)** ventral, **(F)** dorsal, **(J)** cross-sectional microvessels in injury group; **(C)** ventral, **(G)** dorsal, **(K)** cross-sectional microvessels in T50 group; **(D)** ventral, **(H)** dorsal, **(L)** cross-sectional microvessels in T100 group. The white arrow indicates the location of the injury.

At the end of the experiment, 12 weeks after cervical spinal cord injury, one rat was randomly selected from each group for assessment of cervical spinal cord microvascular perfusion; it was placed in the micro-CT device to scan the central part of the injury. The results of the three-dimensional reconstruction of cervical spinal cord microvessels are shown in Figure 7. Notably, vascular density was significantly reduced in the injury group, particularly in terms of the microvessels. Vascular density was also significantly reduced in the T50 group, more obviously than in the injury group; however, the number of microvessels was not significantly reduced in the T100 group. Because of the small number of rats in each group, statistical analysis could not be performed. Therefore, microvascular changes were analyzed by microvascular immunohistochemical staining.

### Rats in the T50 and T100 Groups Preserved Higher Vascular Density in Immunohistochemical Staining Evaluation

**FIGURE 8:**
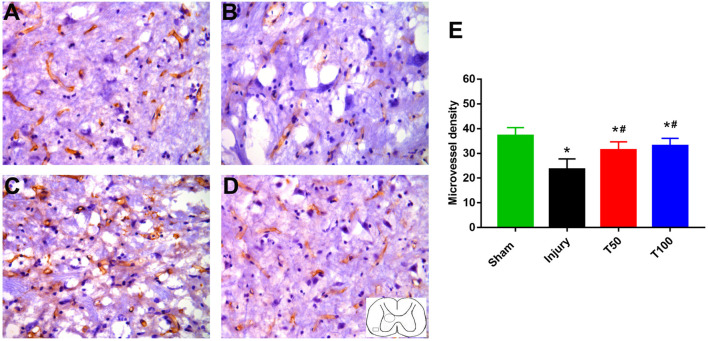
CD34 immunohistochemical staining. **(A)** Sham group; **(B)** injury group; **(C)** T50 group; **(D)** T100 group; **(E)** statistical results of microvascular density showed that MVD of injury group, T50 group and T100 group were significantly lower than Sham group, and the MVD of T50 group and T100 group were significantly higher than injury group. *P < 0.05, comparison between each group and sham group; ^#^*P* < 0.05, comparison between T50 group or T100 group and injury group.

CD34 is a specific protein marker of vascular endothelial cells, and the density of blood vessels in tissues can be determined by CD34 immunohistochemical staining. We stained cervical spinal cord microvessels using this method to determine changes in microvessels among groups. At 12 weeks after spinal cord injury, the microvascular density in the injury, T50, and T100 groups was 23.50 ± 1.75, 31.33 ± 1.36, and 33.00 ± 1.27, respectively. The microvascular density was significantly lower in the injury, T50, and T100 groups compared with the sham group. Moreover, vascular density was significantly higher in the T50 and T100 groups than in the injury group (*P* = 0.0054 and *P* = 0.0013, respectively) (Figure 8).

### Rats in the T50 and T100 Groups Preserved a Greater Parenchyma and Gray Matter in Eriochrome Cyanine Staining Evaluation

**FIGURE 9:**
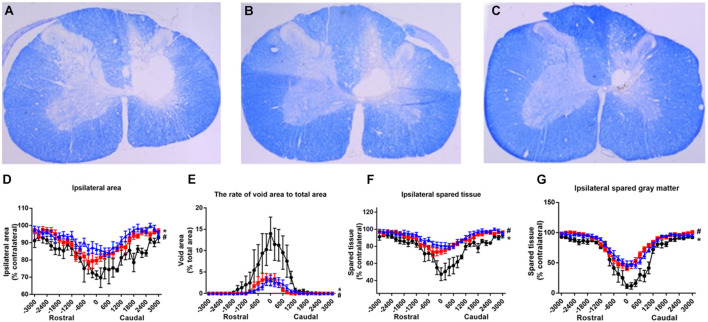
EC staining results. **(A)** Epicenter of injury group; **(B)** epicenter of T50 group; **(C)** epicenter of T100 group; **(D)** percentage of ipsilateral side area relative to contralateral side; **(E)** the percentage of void area relative to total spinal cord area; **(F)** percentage of ipsilateral spared parenchyma relative to the total area of contralateral side; **(G)** percentage of ipsilateral side gray matter relative to contralateral side gray matter. *P < 0.05, comparison between T50 group and injury group; ^#^*P* < 0.05, comparison between T100 group and injury group.

Each set of slices was separated by 200 μm to observe continuous changes from the center to both ends of the lesion. Damage was mainly seen on the ipsilateral side, in the form of spinal cord atrophy, cavity formation, and a reduction in the gray matter area; the contralateral side was generally intact and exhibited normal morphology. Slice staining showed that the injured area was significantly larger in the T50 and T100 groups than in the injury group (Figure 9D). The lesion epicenter was largest in the injury group (Figure 9A), while the lesion area was significantly smaller in the T50 and T100 groups (Figures 9B,C) than in the injury group (Figure 9E). The areas of residual parenchyma and residual gray matter (Figures 9F,G) were significantly larger in the T50 and T100 groups than in the injury group.

## Discussion

This study showed that treatment with trihydroxyethyl rutin could promote motor function recovery after cervical spinal cord injury in rats. The treatment also promoted SEP and MEP recovery, maintained microvascular density, reduced spinal cord atrophy severity and lesion area size, and retained more of the spinal cord parenchyma.

Cervical spinal cord injuries comprise more than 50% of all spinal cord injuries encountered in clinical practice (Khan et al., 2009). Although most cases of spinal cord injury occur in the cervical spinal cord, many studies use animal models of thoracic spinal cord injury (Basso et al., 1996). In recent years, research on cervical spinal cord contusion models has received increasing attention. These models more closely reflect spinal cord trauma in humans (Kong et al., 2017; Huang et al., 2018); notably, cervical spinal cord contusion models differ from full injury models in terms of the histology on the ipsilateral side. Moreover, affected animals have greater tolerance to hemilateral injury, which leads to persistent functional forelimb defects on the ipsilateral side; these models are convenient for studies on feeding and nursing, and for comparative observations. The Instron E1000 testing machine used in this study has motor and electromagnetic control technologies allowing accurate measurement of limb displacement, “bruising speed,” and strike force. The Instron E1000 testing machine has greatly improved the consistency and accuracy of spinal contusion models. The cervical spinal cord hemi-contusion model established in this study was based on a previously published method (Liu et al., 2019a).

As noted in the Introduction, rutin is a flavonoid with multiple beneficial biological effects. Hao et al. (2016) reported that rutin can improve neurological function in rats with subarachnoid hemorrhage; it can also reduce blood-brain barrier permeability, lower cerebral water content, and prevent cerebral cortical neuron death. In rat models of cerebral hemorrhage and ischemia-reperfusion, rutin can alleviate bleeding or reduce the area of cerebral infarction; it also reduces deficits in short-term memory and motor coordination (Khan et al., 2009; Jang et al., 2014; Mondello et al., 2015; Li et al., 2017; Liu et al., 2018). Trihydroxyethyl rutin is a modified form of rutin that is more easily absorbed by the body. However, its therapeutic effects for cervical spinal cord injury have not been investigated.

Previous reports have indicated that rutin can improve behavior in rats with cerebral hemorrhage, cerebral ischemia, brain injury, and other types of injury (Khan et al., 2009; Jang et al., 2014). We treated our C5 hemi-contusion model rats with trihydroxyethyl rutin at 50 or 100 mg/kg; improved grip strength and frequency of forelimb use were seen in both treatment groups. SEPs and MEPs are quantitative parameters enabling objective assessment of sensory and motor pathway integrity, and thus nervous function recovery (Huang et al., 2018). In this study, we used neuroelectrophysiological monitoring to observe continuous dynamic changes in SEPs and MEPs in hemi-cervical spinal cord contusion rats, from a preoperative time point to 12 weeks postoperatively. The results showed that the SEP and MEP amplitudes were better maintained, and the latency was lower, in both treatment groups than in the injury group.

An intact vascular structure is important for normal function in the cervical spinal cord. Spinal cord injury is accompanied by obvious changes in vascular structure; microvessel damage leads to further neurological dysfunction (Melissano et al., 2015). A clear understanding of spinal cord microvascular events (e.g., loss of microcirculation, destruction of the blood-spinal barrier, loss of structural tissue, death of endothelial cells, and vascular remodeling) (Dong et al., 2016) is important because vascular injury can exacerbate the effects of secondary injury after the initial spinal cord injury (Figley et al., 2014). Because the pathological process that occurs during cervical spinal cord injury is closely related to microvascular structure damage (Wu et al., 2018), microvascular changes can serve as an indirect measure of the efficacy of treatment strategies; here, we used blood perfusion analysis and three-dimensional reconstruction of cervical spinal cord microvessels to characterize the microvascular morphology after trihydroxyethyl rutin treatment in rats with cervical spinal cord injury. We then performed CD34 immunohistochemical staining to investigate whether trihydroxyethyl rutin can help maintain microvascular density after spinal cord injury.

MRI is an important clinical modality to determine the site and degree of spinal cord injury in the coronal, sagittal, and transverse planes. Diffusion-weighted functional imaging can be used to evaluate the status of residual nerve fibers after spinal cord injury (Li et al., 2015; Moon et al., 2018). This study used 7.0 T MRI to scan white and gray matter structures in rats, and demonstrated that trihydroxyethyl rutin can significantly reduce the cervical spinal cord injury area in the coronal, sagittal, and transverse planes; these findings were consistent with subsequent Eriochrome cyanine staining results, thus confirming the neuroprotective effect of trihydroxyethyl rutin in rats with cervical spinal cord injury.

A notable limitation of this study was that we did not investigate the mechanism underlying the neuroprotective effect of trihydroxyethyl rutin in our model of cervical spinal cord injury; this will be investigated in future studies.

## Conclusion

Trihydroxyethyl rutin has a robust neuroprotective effect in rats with cervical spinal cord injury; it can improve limb motor function and neural electrophysiological parameters, while maintaining microvascular density and reducing the lesion area. Trihydroxyethyl rutin may be a new option for clinical treatment of spinal cord injury, but the mechanism underlying its neuroprotective effect requires additional research.

## Data Availability Statement

The original contributions presented in the study are included in the article/supplementary material, further inquiries can be directed to the corresponding author/s.

## Ethics Statement

The animal study was reviewed and approved by the Southern Medical University Institutional Animal Care and Use Committee. All animal procedures were conducted in accordance with the National Institutes of Health guidelines for the care and use of experimental animals.

## Author Contributions

XLW and QZ conceived and supervised the study. YL and QZ designed the experiments. YL, QL, ZY, RL, ZPH, ZCH, JHL, XHW, and JYL performed the experiments. YL, QL, and ZY analyzed the data. YL wrote the manuscript. All authors read and approved the manuscript.

## Conflict of Interest

The authors declare that the research was conducted in the absence of any commercial or financial relationships that could be construed as a potential conflict of interest.

## Publisher’s Note

All claims expressed in this article are solely those of the authors and do not necessarily represent those of their affiliated organizations, or those of the publisher, the editors and the reviewers. Any product that may be evaluated in this article, or claim that may be made by its manufacturer, is not guaranteed or endorsed by the publisher.
